# Surgical Site Infection Risk Factors in Hip Arthroplasty for Transcervical Femoral Neck Fractures

**DOI:** 10.7759/cureus.63916

**Published:** 2024-07-05

**Authors:** Héctor E Arriaga-Cazares, Jose Pablo Rodriguez-Lopez, Fernando Ancira-Gonzalez, Sergio Charles-Lozoya

**Affiliations:** 1 Research, Unidad Médica de Alta Especialidad (UMAE) Hospital de Traumatología y Ortopedia No. 21, Monterrey, MEX; 2 Orthopaedics, Unidad Médica de Alta Especialidad (UMAE) Hospital de Traumatologia y Ortopedia No. 21, Monterrey, MEX

**Keywords:** surgical site infection (ssi), total hip arthroplasty (tha), blood transfusion safety, inpatient hip fracture, packed red blood cell transfusion

## Abstract

Background

Hip fracture patients often experience surgical site infections (SSIs) as a major infectious complication after undergoing total hip arthroplasty (THA), which can lead to extended hospital stays, increased mortality, and higher healthcare costs. This study aimed to determine the incidence of SSI and identify the risk factors associated with it after THA.

Objective

This study aimed to explore the correlation between blood transfusion along with other factors and the occurrence of SSIs in postoperative patients who underwent THA for transcervical femoral neck fractures.

Methods

We conducted a retrospective analysis by reviewing the medical records of patients aged 60-80 years who underwent surgery for hip fractures at the Unidad Médica de Alta Especialidad Hospital de Traumatología y Ortopedia No. 21 in Monterrey, Mexico, between January 2020 and January 2021. We analyzed potential risk factors such as age, sex, transfusion necessity, preoperative hemoglobin levels, history of diabetes mellitus, arterial hypertension, and end-stage chronic disease. Data are presented as numbers and percentages, and statistical analyses were performed using IBM SPSS Statistics for Windows, Version 28.0 (Released 2021; IBM Corp., Armonk, New York, United States).

Results

The study included 87 patients, of whom 55 (63%) were women with an average age of 73 years. SSIs were identified in 12 (13.8%) patients. Among those with infections, nine (75%) had a history of blood transfusion (p=0.05). Diabetes, hypertension, and chronic kidney disease also increased the risk for infection. There was no association with gender, age, American Society of Anesthesiologists (ASA) risk, and preoperative hemoglobin.

Conclusions

We found a heightened risk of SSI in patients with a history of blood transfusions, emphasizing the need for careful consideration and monitoring during the perioperative period. Additionally, patients with comorbidities such as diabetes, hypertension, and chronic kidney disease were more susceptible to SSI, underscoring the importance of preoperative assessment and targeted preventive measures. Further research and collaboration are needed to refine strategies for mitigating SSI risk factors and optimizing healthcare resource utilization.

## Introduction

Surgical site infection (SSI) denotes an infection affecting the incision, organ, or space post-surgery [1]. Typically, these infections are caused by the host pathogens. However, among patients hospitalized for more than five days, hospital-borne infection becomes the predominant culprit [2].

Hip fractures are a common cause of orthopedic hospitalization. Its incidence increases with age, with 90% of cases occurring in individuals aged >50 years, with an average age of 80 years, particularly in women. Falls and trips account for most cases, with an annual risk of 4%. In Mexico, fractures in the femoral neck and transtrochanteric region account for 90% of all hip fractures [3].

Total hip arthroplasty (THA) is commonly performed for femoral neck fractures or advanced inflammatory/degenerative arthritis [4]. Anemia triggered by blood loss is a relatively frequent occurrence after hip arthroplasty, and standard treatment involves blood transfusion from an allogeneic donor [5]. In 2017, a study revealed that 2-70% of patients received blood transfusions during or after post-arthroplasty surgery [6].

Browne et al. [7] highlighted the link between blood transfusion and increased heightened postoperative morbidity and mortality. It has been suggested that the risk of SSI corresponds to the transfusion dosage. Moreover, comparing autogenous to allogeneic transfusions reveals that the heightened infection risk pertains only to allogeneic transfusions [8].

SSI is a critical complication of THA, affecting 0.5-3% of patients. If this complication is severe, it can result in patients often requiring prosthesis removal, prolonged antimicrobial therapy, and a month-long reimplantation delay [9].

This study aimed to investigate whether blood transfusion, among other variables, correlates with an elevated occurrence of SSI in postoperative total hip replacement patients following transcervical femoral neck fractures.

## Materials and methods

We conducted a retrospective analysis by reviewing the medical records and surgical reports. We collected cross-sectional data from patients aged 60-80 years who underwent THA for hip fractures at the Unidad Médica de Alta Especialidad Hospital de Traumatología y Ortopedia No. 21 in Monterrey, Mexico, between January 2020 and January 2021. Patients with a history of fractures in the lower extremities, inflammatory arthropathies, hematological conditions affecting coagulation, or immune disorders were excluded from the study.

The variables analyzed were age, sex, necessity for transfusion, preoperative hemoglobin levels, history of diabetes mellitus, arterial hypertension, and end-stage chronic disease. The diagnosis of SSI was made based on criteria including a positive wound culture indicating the presence of organisms, abnormal swelling around the surgical site, localized heat, erythema, and pain.

This study was performed in accordance with the guidelines of the Declaration of Helsinki and its later amendments or comparable ethical standards. The study protocol was approved by our hospital's Research Ethics Committee and Local Health Research Committee (approval number: R-2021-1903-017).

Statistical analysis

Data are presented as numbers and percentages. The study groups were compared using the chi-squared test. Quantitative variables were compared using the t-test. Results were considered statistically significant at P<0.05. Statistical analyses were performed using IBM SPSS Statistics for Windows, Version 28.0 (Released 2021; IBM Corp., Armonk, New York, United States).

## Results

A total of 87 patients were included in the evaluation, of which 55 (63%) were women. The mean age was 73.4 years. Among the subjects included in the study, 12 (13.8%) had an SSI, of which nine (75%) had a deep infection and three (25%) had a superficial site infection. Among the participants, 43 (49%) required blood transfusion. The most prevalent comorbidities were arterial hypertension (55.2%), diabetes mellitus (38%), and chronic kidney disease (16%). Sixty-five (74.7%) had an American Society of Anesthesiologists (ASA) score of 2. The mean hemoglobin level among the patients was 12.5 mg/dl (Table 1). 

**Table 1:** Demographic characteristics of study participants SSI: surgical site infection; HTN: hypertension; CKD: chronic kidney disease; DM: diabetes mellitus; ASA: American Society of Anesthesiologists

	N (%)
Female	55 (63.2)
Age, years (median, IQR)	74 (67-81)
Required transfusion	44 (50.6)
SSI	12 (13.8)
Comorbidities	
HTN	48 (55.2)
CKD	16 (18.4)
DM	38 (43.7)
ASA classification	
1	5 (5.7)
2	65 (74.7)
3	17 (19.5)
Pathogenic agents	
Staphylococcus epidermidis	5 (41)
Staphylococcus aureus	3 (25)
Escherichia coli	1 (8)
Pseudomonas aeruginosa	1 (8)
Serratia marcescens	1 (8)
Enterococcus faecalis	1 (8)

Patients with diabetes mellitus and chronic kidney disease exhibited an increased risk of SSI compared to those without these conditions (p=0.003 for diabetes mellitus; p=0.007 for chronic kidney disease). Additionally, the analysis revealed that patients who underwent blood transfusions faced a significantly increased risk of SSI (p=0.026) compared to those who did not receive transfusions (see Table 2).

**Table 2:** Factors associated with SSIs in 87 patients undergoing arthroplasty for transcervical femur fracture SSI: surgical site infection; HTN: hypertension; CKD: chronic kidney disease; DM: diabetes mellitus; ASA: American Society of Anesthesiologists

	With SSI (n=12)	Without SSI (n=75)	p
Age	75.4±8.18	73.1±10	0.78
Female	4 (33)	51 (68)	0.026
Preoperative hemoglobin	12.2±1.87	12.3±1.67	0.3
Blood transfusion	9 (75)	34 (45.5)	0.05
DM	10 (83.3)	28 (37.3)	0.003
HTN	10 (83.3)	38 (50.7)	0.033
CKD	6 (50)	10 (13.3)	0.007
ASA risk			0.60
1	0	5 (6.7)	
2	9 (75)	56 (74.7)	
3	3 (25)	14 (18.7)	

## Discussion

In this retrospective study, we analyzed data collected over a one-year period at our medical center to examine the factors contributing to postoperative SSIs. Our findings indicated that blood transfusion, timing of transfusion, and presence of diabetes were significant factors predisposing patients to postoperative SSIs.

Femoral neck fractures are a prevalent type of fracture in orthopedics, particularly among elderly individuals. Surgical intervention is the typical treatment approach for helping patients with femoral neck fractures to resume their daily activities as soon as possible. However, SSIs are a common postoperative complication for patients with femoral neck fractures [10].

The incidence of postoperative infections was 14%, which surpassed the infection rates reported in the existing literature. Notably, Marom et al. [11] reported a mere 3% infection incidence following surgery in Israel, whereas Alegre-Rico and Orozco [12] documented an 8% SSI rate in a Mexican study. Our analysis suggests that the higher infection rate in our population may be attributed to the advanced age and increased prevalence of comorbidities among our patients. These factors likely played a significant role in influencing the outcomes and highlight the importance of considering patient demographics and health status when assessing postoperative infection risks.

In our study, patients who received blood transfusions showed a 69% rate of SSIs, a statistic in line with the findings of Everhart et al. [13], whose research indicated that 61% of SSI cases involved patients who had undergone blood transfusions. These consistent findings suggest a need for heightened awareness and targeted interventions in cases involving transfused patients. Understanding and addressing the factors contributing to this increased risk can be crucial for refining surgical practices, optimizing patient outcomes, and ultimately reducing the incidence of SSIs in this specific patient population.

Our study adds valuable insights to the understanding of risk factors for SSIs. Consistent with the findings of Resende et al. [14], our data reaffirms the association between certain medical conditions, specifically, diabetes mellitus, chronic kidney disease, congestive heart disease, and arterial hypertension, and an increased risk of SSIs. This alignment with existing research highlights the robustness of these identified risk factors across different patient cohorts. Importantly, our study departs from the conclusions drawn by Kong et al. [15] in 2016, as we did not observe a significant link between ASA risk scores and an elevated risk of infection. This discrepancy emphasizes the complexity of the relationship between patient health indicators and SSIs, underscoring the need for ongoing research to refine our understanding and inform targeted preventive measures.

The patterns observed in our study echo trends identified in other surgical realms, particularly in cardiac surgery, where transfusions have been linked to heightened risks of mortality and morbidity, even in patients with lower hematocrit levels [16]. This parallel underscores the potential systemic impact of blood transfusions on patient outcomes across diverse surgical procedures. However, promisingly, our findings align with emerging strategies in mitigating the associated risks. Notably, the utilization of antifibrinolytic agents has demonstrated efficacy in reducing the complications linked to blood transfusions in institutional settings [17]. This points towards a potential avenue for intervention and improvement in patient care. As we continue to unravel the complexities of transfusion-related risks, the exploration of targeted interventions, such as antifibrinolytic agents, holds promise for optimizing patient safety and outcomes in various surgical contexts. Further research and implementation of evidence-based practices can contribute to refining surgical protocols and advancing patient care in the face of these challenges.

The absence of statistical significance in factors traditionally considered as contributors to SSIs, such as surgical risk, preoperative hemoglobin level, age, and sex, challenges prevailing assumptions and highlights the nuanced nature of postoperative complications. While our findings may redefine the risk profile specifically in the context of femoral neck fractures, they underscore the importance of questioning established paradigms in surgical research. These unexpected results prompt a critical reevaluation of risk assessment models, emphasizing the need for a tailored and context-specific approach to understanding the dynamics of SSIs. As we continue to unravel the intricacies of postoperative complications, the quest for a more comprehensive understanding remains ongoing.

Limitations

One primary limitation of our study is its retrospective and uncontrolled design. The inherent nature of retrospective studies raises the possibility of selection bias and confounding variables, limiting our ability to establish definitive causal relationships between the identified factors and postoperative SSIs. Additionally, the reliance on data collected over a one-year period at a single medical center may introduce limitations related to generalizability, as the findings may not fully represent the broader population.

Furthermore, the focus on femoral neck fractures in orthopedics, while allowing for in-depth analysis within a specific context, may limit the generalizability of our findings to other surgical procedures or patient populations. The prevalence of femoral neck fractures among elderly individuals and the associated postoperative complications may introduce a unique set of factors that may not be applicable to different surgical scenarios.

The generalizability of our findings may be influenced by the specific patient demographics and characteristics of our study population. The higher incidence of postoperative infections in our cohort, compared to rates reported in the literature, suggests the need for caution when extrapolating our results to populations with different age distributions or comorbidity profiles. Consequently, the applicability of our conclusions to diverse surgical settings and patient groups should be approached with careful consideration.

While our study provides valuable insights into the risk factors for SSIs in the context of femoral neck fractures, the generalizability of these findings to broader surgical practices necessitates further research across various surgical specialties and patient populations. Prospective, controlled studies involving larger and more diverse cohorts will be essential to enhance the external validity of our observations and contribute to the development of universally applicable preventive strategies for postoperative complications.

## Conclusions

Blood transfusion and presence of diabetes were significant factors predisposing patients to postoperative SSIs. The incidence of postoperative infections was 14%, which surpassed the infection rates reported in existing literature. Other risk factors such as diabetes, hypertension, and chronic kidney disease also showed a correlation.

The study highlights the importance of considering patient demographics and health status when assessing postoperative infection risk, emphasizing the need to develop strategies to minimize instances of blood transfusion.
